# Evaluation of renal values during treatment for heartworm disease in 27 client-owned dogs

**DOI:** 10.1186/s13071-023-05779-0

**Published:** 2023-06-09

**Authors:** C. Autumn M. Vetter, Alison G. Meindl, Bianca N. Lourenço, Michael Coyne, Corie Drake, Rachel Murphy, Ira G. Roth, Andrew R. Moorhead

**Affiliations:** 1 Department of Small Animal Medicine and Surgery, College of Veterinary Medicine, University of Georgia, Athens, GA USA; 2 Department of Clinical Sciences, College of Veterinary Medicine, Colorado State University, Fort Collins, CO USA; 3 IDEXX Laboratories, Westbrook, ME USA; 4 Department of Infectious Diseases, College of Veterinary Medicine, University of Georgia, Athens, GA USA

**Keywords:** Canine, *Dirofilaria immitis*, Dirofilariasis, Kidney disease, Melarsomine, Symmetric dimethylarginine

## Abstract

**Background:**

Canine heartworm disease (CHD) caused by *Dirofilaria immitis* remains a common preventable disease with increasing incidence in some parts of the USA. The treatment guidelines of the American Heartworm Society (AHS) currently recommend monthly macrocyclic lactone administration, 28 days of doxycycline given orally every 12 h and three injections of melarsomine dihydrochloride (1 injection on day 2 of treatment followed 30 days later by 2 injections 24 h apart). Minocycline has also been utilized when doxycycline is unavailable. The systemic effects of CHD, which particularly impact cardiac and renal function, have been described, with infected dogs often experiencing renal damage characterized by an increase in serum concentrations of renal biomarkers. Although the AHS treatment protocol for CHD has been shown to be safe and effective in most cases, the potential for complications remains. No study as of yet has evaluated changes in symmetric dimethylarginine (SDMA), a sensitive marker of renal function, during treatment for CHD. The purpose of the present study was to evaluate renal function in dogs by measuring serum creatinine and SDMA concentrations during the adulticide treatment period.

**Methods:**

Serum creatinine and SDMA concentrations were measured in 27 client-owned dogs affected by CHD at the following time points: prior to starting doxycycline or minocycline therapy (baseline), during doxycycline or minocycline therapy (interim), at the time of the first dose of melarsomine (first dose), at the time of the second dose of melarsomine (second dose) and at the dog’s follow-up visit after treatment, occurring between 1 and 6 months after completion of therapy (post-treatment). Concentrations of creatinine and SDMA were compared between time points using a mixed effects linear model.

**Results:**

Mean SDMA concentrations following the second dose of melarsomine were significantly lower (−1.80 ug/dL, *t*-test, df = 99.067, *t* = −2.694, *P*-Value = 0.00829) than baseline concentrations. There were no other statistically significant differences in the concentration of either biomarker between the baseline and the other time points in CHD dogs undergoing treatment.

**Conclusions:**

The results suggest that the current AHS protocol may not have a substantial impact on renal function.

**Graphical Abstract:**

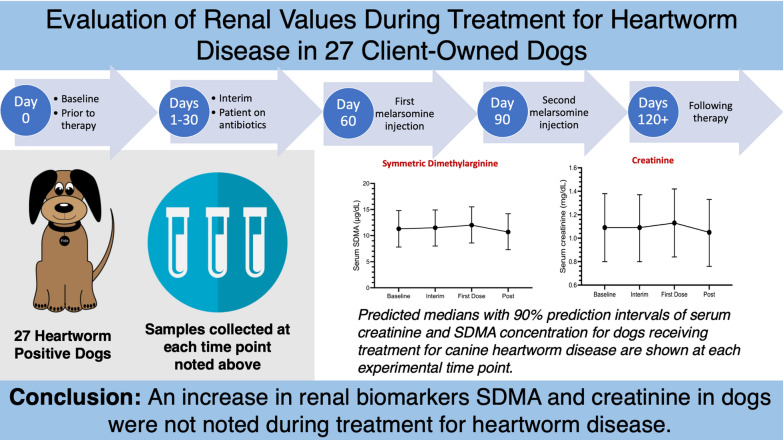

## Background

Canine heartworm disease (CHD) caused by *Dirofilaria immitis* remains a common, preventable disease, the incidence of which is increasing in some parts of the USA despite the availability of prevention measures [1]. Treatment protocols for CHD vary according to geography, client compliance and veterinarian recommendations [2]. The American Heartworm Society (AHS) currently recommends treatment with macrocyclic lactone administered at monthly intervals, 28 days of twice-daily doxycycline 10 mg/kg body weight (BW) administered orally, and three deep intramuscular injections of melarsomine dihydrochloride at days 60, 90 and 91 of treatment [3]. This protocol has been shown to be effective and safe for the treatment of adult heartworms in dogs with CHD [3].

Although treatable, CHD can impact organ function in dogs before and after therapy. Evaluation of cardiac biomarkers, specifically cardiac troponin I (cTnl), myoglobin and D-dimer, has suggested the presence of myocardial injury and heart failure in both acute and chronic CHD infections [4]. Renal damage and dysfunction have also been demonstrated with an increase in specific serum biomarkers, including serum urea nitrogen and creatinine, and the development of proteinuria in some dogs [5]. In one study, 19% of dogs with CHD had proteinuria identified during the pre-treatment diagnostic evaluation, with 4.2% of the dogs having elevated concentrations of either serum creatinine or symmetric dimethylarginine (SDMA) [5].

Although shown to be overall safe and effective, adulticide treatment of CHD can result in several complications during the treatment period, regardless of the treatment protocol. These complications include congestive heart failure, thromboembolic disease and kidney dysfunction [6]. One study demonstrated that blood urea nitrogen (BUN) and creatinine concentrations remained within the respective reference intervals during the AHS-recommended treatment period, suggesting that renal function, although impacted by the disease process, is not further impacted during therapy for CHD [7].

Creatinine has been the primary biomarker for glomerular filtration rate (GFR) and, therefore, kidney function for decades, but it is susceptible to extra-renal factors, including changes in muscle condition of the animal [8]. A further limitation of creatinine as a biomarker is that its concentration does not increase above the reference interval until roughly 75% of the functional nephrons have been lost [9–11], thereby limiting its usefulness for early detection of renal dysfunction. Another common marker of renal function is urine concentrating ability, or urine specific gravity (USG). However, impaired urinary concentrating ability is not observed until roughly two-thirds of functional nephrons have been lost [12, 13]. SDMA is a biomarker of GFR that has been shown to increase above its reference interval earlier than creatinine in cases of renal impairment, with one study reporting increased SDMA concentration approximately 17 months prior to increased blood creatinine concentration [14]. As such, SDMA has recently been included in the chronic kidney disease (CKD) staging system set forth by the International Renal Interest Society [15].

Along with the ability to detect smaller decreases in renal functional mass, SDMA concentrations are subjected to fewer extra-renal factors, as these are not affected by lean body mass [16]. Additionally, SDMA was found to be evaluated in dogs with heartworm disease, in which its concentrations were elevated in both asymptomatic and symptomatic dogs compared to heartworm-negative controls even though creatinine concentrations were not significantly different between the groups [17]. Because renal function must be significantly compromised to impact creatinine concentrations, the impact of the AHS treatment protocol on renal function is not completely known. One study that evaluated the renal function of dogs undergoing therapy using the AHS protocol—i.e. by measurement of creatinine and BUN concentrations as well as urinary protein-to-creatinine ratio—found no changes in these parameters during therapy [7]. To date, however, there have been no studies evaluating SDMA in dogs undergoing treatment for CHD using the AHS protocol.

The purpose of the present study was to evaluate renal function in dogs by measuring SDMA and creatinine concentrations during the adulticide treatment period. Specifically, we sought to determine whether SDMA or creatinine concentrations increase from baseline during treatment with doxycycline or minocycline or at the first or second dose of melarsomine in subsequent visits.

## Methods

### Study population

This retrospective study was performed using banked serum samples collected from 27 client-owned dogs infected by *D. immitis* that participated in an unrelated prospective study evaluating the efficacy of doxycycline and minocycline as part of the heartworm treatment protocol [18]. The dogs included in that study lived in an endemic area, with 2.17% of the population testing positive for CHD in 2021 (range: 1–27%)[19], and were presented to the university primary care center for treatment of CHD following diagnosis by their local primary care veterinarian. Dogs were recruited for that previous study following diagnosis.

The diagnosis of heartworm infection was established based on the presence of circulating antigen in serum samples, as determined by using the DiroCHEK® Heartworm Antigen Test Kit (Synbiotics Corp., Zoetis Inc., Kalamazoo, MI, USA). The serum samples were also evaluated by the modified Knott test to determine the presence or absence of microfilaria, as previously described [18]. After diagnosis, the dogs were randomized to receive 10 mg/kg BW or 5 mg/kg BW of either doxycycline or minocycline as the treatment for *Wolbachia* (various brands used based on availability). All dogs received once-monthly ivermectin/pyrantel (Heartgard Plus®; Boehringer Ingelheim, Duluth, GA, USA) for a total of 6 months, 28 days of tetracycline treatment according to randomization and the three-dose protocol of 2.5 mg/kg BW melarsomine dihydrochloride for the treatment of heartworm disease based on the AHS guidelines [3]. Dogs received either Immiticide® (Merial Limited, Duluth, GA, USA) or Dirobran™ (Zoetis Inc.) according to the availability of these products.

Dogs were followed throughout the course of treatment, with blood samples collected and banked at the various time points described in section Sample collection and storage. The collection of samples used in the present study was approved by the University of Georgia’s Clinical Research Committee, the Hospital Board and the University of Georgia Research Foundation prior to the start of the original study [18].

### Sample collection and storage

**Fig. 1:**
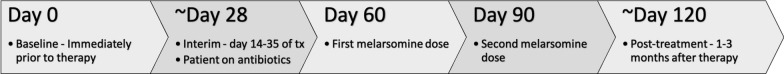
Overview of time points for the study from which banked samples used in the present study were collected. tx, Treatment

Serum samples were collected at each of the following time points, as previously described (Fig. 1; [18]): (i) immediately prior to beginning doxycycline or minocycline therapy (baseline); (ii) around day 28 (range: day 14–35) of treatment with doxycycline or minocycline (interim period); (iii) at the time of administration of the first melarsomine injection (first dose period); (iv) at the time of administration of the second melarsomine injection (second dose period); and (v) around day 120 (range: day 120–180 [months 1–3]) following the dogs’ third dose of melarsomine (post-treatment period)

For each dog, multiple blood samples were collected on separate occasions (as outlined above) during the interim, first dose, second dose, and post-treatment periods. After blood collection, blood was allowed to clot, and serum was separated out. Serum samples were stored at − 80 °C until they were shipped to IDEXX laboratories (Westbrook, ME, USA) on dry ice for further analysis.

### Creatinine and SDMA

Serum creatinine concentrations were determined by a colorimetric method, namely Jaffe’s reaction using picrate at alkaline pH, with changes in color measured on a Beckman Coulter spectrometer (Beckman Coulter, Inc., Diagnostics Division, Brea, CA, USA). Serum SDMA concentrations were determined using a commercially available high-throughput immunoassay (IDEXX SDMA® Test; IDEXX Laboratories, Inc., Westbrook, ME, USA).

### Statistical analysis

The primary research questions explored in this study focused on changes in serum SDMA and creatinine concentrations between the various time points during treatment and a baseline measurement taken before treatment. For each dependent variable (serum SDMA and creatinine concentrations), a mixed effects linear model was fit with a fixed effect of time point and random intercepts for each animal. Hypotheses were tested by constructing the appropriate contrasts of model parameter estimates.

In the study for which the samples were originally collected, dogs received two different antibiotics, doxycycline and minocycline, at two different dosages: 5 or 10 mg/kg BW. Data pertaining to doxycycline and minocycline dosage were merged, and the SDMA and creatinine results collected in the present study were used to test the secondary hypothesis of whether dosage affects the change in analyte concentration between the baseline and interim time points. Mixed effects linear models were fit for each analyte with fixed effects time point and dosage, with dosage nested within time point, and random intercepts for each animal.

Statistical analyses were performed using commercially available software: R software environment (2022; https://www.R-project.org/) and GraphPad Prism for Mac, version 9.4.1 (GraphPad Software, Inc., La Jolla, CA, USA). A significance level of 0.05 was used for all analyses.

## Results

Of the data for this study are comprised of longitudinal results from 27 dogs receiving treatment for heartworm disease. The number of observations (categorized as mentioned in section Methods) for each dog varied between 7 and 18, with a median of 10 observations.

The 27 studied dogs were of varying breed, 12 were female and 15 were male and their ages ranged from 1 to 8 years (mean age: 4 years). Seven dogs were treated with doxycycline 10 mg/kg BW twice daily, six were treated with doxycycline 5 mg/kg BW twice daily, six were treated with minocycline 10 mg/kg BW twice daily and eight were treated with minocycline 5 mg/kg BW twice daily. Throughout the course of treatment, there were no significant changes in body weight. Staging diagnostic results did not have any significant bearing on the study at hand or on patient outcome during the course of treatment and are not reported.

**Fig. 2:**
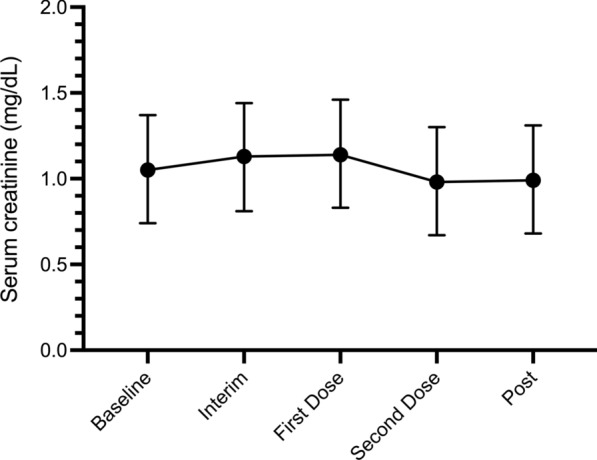
Predicted medians with 90% prediction intervals of serum creatinine concentrations for dogs receiving treatment for canine heartworm disease (*n* = 27) are shown at each experimental time point. Baseline evaluation occurred before treatment; interim evaluation occurred from day 14 to day 35 of treatment while the dogs were receiving antibiotics; first dose evaluation occurred on day 60 at the time of the first melarsomine injection; second dose occurred on day 90 at the time of the second melarsomine injection; and post-treatment evaluation occurred between months 1 and 3 following the dogs’ third dose of melarsomine (Post)

**Fig. 3:**
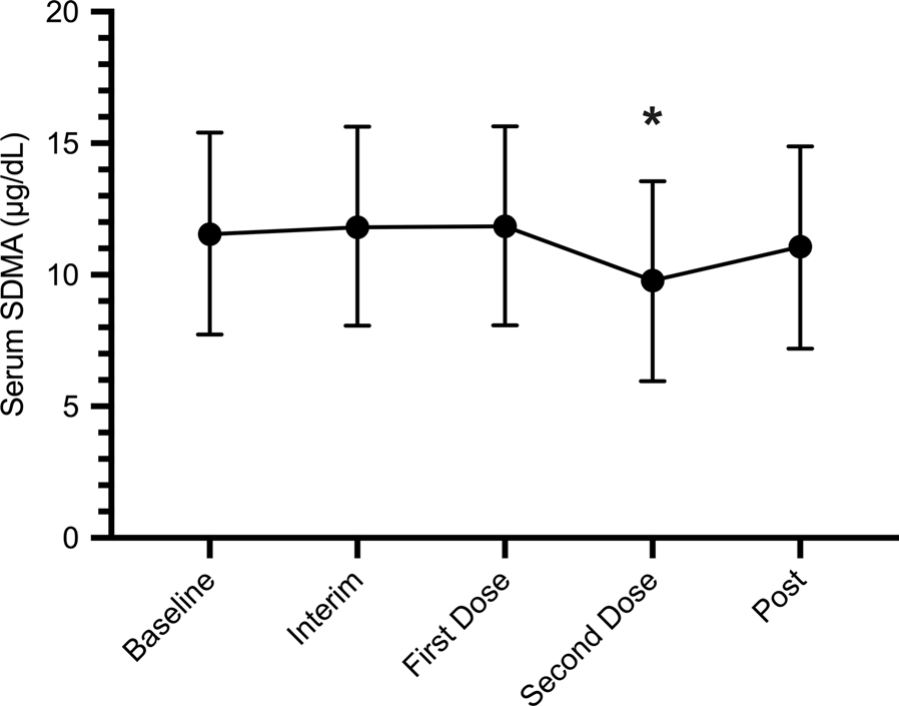
Predicted medians with 90% prediction intervals of serum SDMA concentration for dogs receiving treatment for canine heartworm disease (*n* = 27) are shown at each experimental time point. See Fig. 2 caption for definition of time period. SDMA, Symmetric dimethylarginine

A significant decrease in SDMA concentrations below the baseline value was observed following the third dose of melarsomine (−1.80 ug/dL, *t*-test, df = 99.067, *t* = −2.694, *P*-Value = 0.0083). There were no other significant changes in serum concentrations of creatinine (*t*-tests Interim: 0.07 mg/dL, df = 95.811, *t* = 1.446, *P*-Value = 0.1515; First dose: 0.09 mg/dL, df = 94.446, *t* = 1.759, *P*-Value = 0.0818; Second dose: −0.07 mg/dL, df = 95.947, *t* = −1.326, *P*-Value = 0.1878; Post-treatment: −0.06 mg/dL, df = 94.363, *t* = −1.176, *P*-Value = 0.2424) or SDMA (t-tests Interim: 0.30 ug/dL, df = 99.020, *t* = 0.474, *P*-Value = 0.6368; First dose: 0.316 ug/dL, df = 96.690, *t* = 0.498, *P*-Value = 0.6194; Post-treatment: −0.481 ug/dL, df = 96.847, *t* = −0.766, *P*-Value = 0.4457) during the course of treatment between the baseline and other timeoints (Figs. 2, 3). Significant differences in SDMA (*t*-test, −1.77 ug/dL, df = 24.180, *t* = −0.934, *P*-Value = 0.3580) or creatinine concentrations (*t*-test, 0.17 ug/dL, df = 27.485, *t* = 0.842, *P*-Value = 0.4069) during the treatment phase (baseline to interim) were also not found for dogs receiving 10 mg/kg BW compared to 5 mg/kg BW of doxycycline or minocycline.

Serum creatinine and SDMA concentrations were above the reference interval (as outlined by the International Renal Interest Society [IRIS] guidelines) at baseline or during treatment for a total of five and two dogs, respectively, with each dog having only one to two instances at which elevations in these biomarkers were noted. All elevations were mild, never exceeding parameters for IRIS stage 2. No dog had elevations of both serum creatinine and SDMA concentrations at any time. The number of dogs for which serum creatinine and SDMA were above the reference interval at each time point during treatment is summarized in Table 1.

**Table 1:** Number of dogs and overall percentage of sample size for which serum creatinine and symmetric dimethylarginine concentrations were above the reference interval at each time point before and during treatment

Time point	Serum creatinine concentration	Serum SDMA concentration
Baseline	½7 (3.70%)	0/27 (0%)
Day 28 of treatment	3/27 (11.11%)	0/27 (0%)
First melarsomine injection	3/27 (11.11%)	2/27 (7.41%)
Second melarsomine injection	0/27 (0%)	0/27 (0%)
Post-treatment	0/27 (0%)	0/27 (0%)

Values in table are presented as the number of dogs at that time point showing an elevation in serum creatinine concentration and/or serum SDMA concentration, with the percentage of total number of dogs in parentheses

*SDMA* Symmetric dimethylarginine

## Discussion

Multiple studies have demonstrated the association between heartworm infection and renal damage, specifically potentiated by immune complex deposition in the glomerular basement membrane after *D. immitis* infection, leading to proteinuria [20–23]. Chronic interstitial nephritis and glomerulosclerosis with amyloidosis have also been described [21, 24–26]. The presence of microfilaria has been thought to be a potentially important factor leading to proteinuria in some studies [27], while the results of other studies suggest that *Wolbachia* may contribute to immune-mediated kidney disease in CHD [24, 27–29]. All dogs in the present study tested microfilaria positive, but since proteinuria was not evaluated, it is unclear whether this positivity played a role in any changes to renal values or specific renal pathology in the present population.

Studies have shown that SDMA has been shown to be a more sensitive marker for monitoring and identifying decreased renal function because of its earlier elevation with excretory renal impairment [30] and the absence of any influence of lean body mass on its concentration [31]. In the present study, a significant decrease in SDMA values was observed following the third dose of melarsomine (−1.80 ug/dL, *t*-test, df = 99.067, *t* = −2.694, *P*-Value = 0.0083), but given that none of the dogs had an elevated SDMA value at baseline, this decrease is clinically insignificant. No other statistically significant changes in serum SDMA (*t*-tests Interim: 0.30 ug/dL, df = 99.020, *t* = 0.474, *P*-Value = 0.6368; First dose: 0.316 ug/dL, df = 96.690, *t* = 0.498, *P*-Value = 0.6194; Post-treatment: −0.481 ug/dL, df = 96.847, *t* = −0.766, *P*-Value = 0.4457) or creatinine (*t*-tests Interim: 0.07 mg/dL, df = 95.811, *t* = 1.446, *P*-Value = 0.1515; First dose: 0.09 mg/dL, df = 94.446, *t* = 1.759, *P*-Value = 0.0818; Second dose: −0.07 mg/dL, df = 95.947, *t* = −1.326, *P*-Value = 0.1878; Post-treatment: −0.06 mg/dL, df = 94.363, *t* = −1.176, *P*-Value = 0.2424) concentrations were documented at any time point during the heartworm treatment protocol, indicating that utilization of ivermectin/pyrantel and doxycycline or minocycline at 5–10 mg/kg BW twice daily (q12) for 28 days, and three injections of melarsomine at 2.5 mg/kg BW per dose did not cause detectable renal dysfunction that could be appreciated by changes in SDMA or creatinine concentrations.

The main limitation of this study was the lack of urine samples available for analysis. Proteinuria has been a significant concern in dogs with CHD in previous studies, with one study showing its presence in about 19% of dogs with heartworm disease [5]. Another study showed membranous glomerulonephritis in five dogs with CHD, with renal histopathology confirming an immune complex form of glomerulonephritis induced by the *D. immitis* infection along the epithelial side of the glomerular basement membrane [20]. Ideally, the present study would have analyzed urinalyses in each dog along with their serum renal values to evaluate proteinuria and urine concentration throughout the course of treatment as well. These data would have provided further insight as to whether any azotemia noted was renal in nature as well as whether treatment affected the kidneys at a glomerular permeability level, as evidenced by an increase in proteinuria. Samples used for the present study were originally collected for a prior study published in 2018 evaluating the efficacy of minocycline as a substitute for doxycycline during the AHS treatment protocol at varying doses [18]. For this reason, only blood samples were available at the studied time points, and no urine was available for analysis. Proteinuria and USG were, therefore, not evaluated.

Another limitation of the present study is the different types and doses of antibiotics administered. Statistical analysis was performed to determine whether there was a significant difference between the four antibiotic groups (5 mg/kg BW minocycline q12h, 10 mg/kg BW minocycline q12h, 5 mg/kg BW doxycycline q12h, 10 mg/kg BW doxycycline q12h), but given the small sample sizes, a difference could not be detected.

Because systemic arterial hypertension is a well-known risk factor for progression of CKD and proteinuria [32–34], in addition to the above parameters, the IRIS kidney guidelines recommend sub-staging renal disease according to severity of hypertension [15]. There are significant associations between CHD and pulmonary hypertension in dogs [35]. One study also describes systemic hypertension in dogs with CHD, with 21.3% of dogs having a systolic blood pressure > 160 mmHg [5]. Including blood pressure measurements at each time point in future studies would be beneficial for complete characterization of systemic factors that might be affecting renal function or contributing to proteinuria, as well as help further characterize associations between CHD and systemic arterial hypertension. Given that the present study was performed retrospectively on banked samples, it was not possible to evaluate systemic blood pressure and urine. The overall sample size was also small.

## Conclusions

Overall, an increase in the renal markers SDMA and creatinine in dogs as a result of heartworm treatment was not observed in this study. Although further research is needed to more fully evaluate the overall effect that treatment of heartworm disease has on renal function, particularly regarding glomerular permeability, the results of the present study indicate that the current AHS guidelines for treatment appear not to significantly impact renal function, as assessed by surrogate markers of GFR, indicating a high margin of safety. There is therefore no immediate indication based on these data to change the current treatment protocol for CHD.

## Data Availability

Not applicable.

## Abbreviations

AHS: American Heartworm Society
CHD: Canine heartworm disease
SDMA: Symmetric dimethylarginine
